# Forsythiaside A Reduces Acetaminophen Hepatotoxic Metabolism by Inhibiting Pregnane X Receptor

**DOI:** 10.3390/molecules30051187

**Published:** 2025-03-06

**Authors:** Sisi Pu, Yangyang Pan, Zuoyang Wang, Huimin Liu, Jianhui Zhang, Qian Zhang, Meng Wang

**Affiliations:** College of Veterinary Medicine, Gansu Agricultural University, Lanzhou 730070, China

**Keywords:** forsythiaside A, pregnane X receptor, acetaminophen metabolism, oxidative stress, endoplasmic reticulum stress, apoptosis

## Abstract

Overdose intake of acetaminophen (APAP) causes liver injury involving hepatic drug metabolism and activation of oxidative stress pathways, and forsythiaside A (FA) has hepatoprotective pharmacological activity, but knowledge of the mechanism of FA treatment for APAP liver injury is still lacking the literature. In this study, we investigated the effects of FA on the pregnane X receptor (*PXR*) by molecular docking and reporter gene assays. In addition, we explored the effects of FA on oxidative stress, endoplasmic reticulum stress (ERS), apoptosis, and hepatic pathology by interfering with *PXR* in ex vivo and in vivo models. The results showed that FA decreased the *PXR* protein expression level and effectively reduced the oxidative stress level in the APAP model. In addition, FA reduced the expression of ERS pathway ProteinkinaseR-likeERkinase (PERK)-translation initiation factor 2 (eIF-2α)-activating transcription factor 4 (ATF4) by inhibiting *PXR*, and at the same time, decreased the expression of apoptotic proteins C/EBP homologous protein (CHOP), Bax, Caspase 3, and Caspase 7, and elevated the expression of apoptosis-suppressing protein Bcl-2, which ultimately treated the hepatic pathology injury of APAP in mice. The present study confirmed that FA improved APAP metabolism by inhibiting *PXR*-mediated CYP1A2 and CYP3A11 and alleviated APAP-induced hepatic impairment by inhibiting hepatic oxidative stress, ERS, and apoptosis.

## 1. Introduction

Drug-induced liver injury (DILI) is a predictable event that occurs when an individual is exposed to toxic doses of certain compounds and is emerging as one of the leading causes of liver health in humans [1]. Acetaminophen (APAP) is a widely used antipyretic analgesic drug worldwide that produces severe hepatotoxicity when overdosed or chronically used [2]. The hepatotoxicity of APAP is mainly caused by its metabolite N-acetyl-p-benzoquinoimide (NAPQI) during the biotransformation process [3]. NAPQI accumulation and glutathione (GSH) depletion after excess APAP enters the CYP450 enzyme (CYP2E1, CYP1A2, CYP3A4) pathway results in excessive reactive oxygen species production, thereby triggering oxidative stress. In addition, the accumulation of peroxides and GSH depletion ultimately lead to endoplasmic reticulum stress (ERS), hepatocyte apoptosis, and even hepatic dysfunction [4].

The pregnane X receptor (*PXR*) is a member of a subfamily of nuclear receptors, which are characterized by their ligand-dependency and their ability to modulate the expression of target genes. From a clinical perspective, approximately 50% of drugs undergo metabolism via the activation of *PXR*-mediated drug-metabolizing enzymes, specifically cytochrome P450 3A11 (CYP3A11) and cytochrome P450 1A2 (CYP1A2). [5]. CYP1A2, CYP2E1, and CYP3A11 (the mouse homologue of human CYP3A4) are the most active CYPs catalyzing the generation of toxic NAPQI from APAP in mice, and previous studies consistently show that induction of these enzymes exacerbates APAP hepatotoxicity. In addition, these CYPs can be regulated by the *PXR*, an important transcriptional regulator of CYP3A11 and CYP1A2. However, mRNA expression of hepatic CYP3A11 and CYP1A2 in mice lacking *PXR* was lower than in the controls [6]. It indicated that *PXR*-deficient mice are less susceptible to APAP-induced liver injury (AILI), which was confirmed by the study of Kristina KW et al. [7].

ERS triggers complex adaptive or apoptosis-promoting signaling defined as the unfolded protein response (UPR) [8]. ProteinkinaseR-likeERkinase (PERK) is a protein kinase distributed in the endoplasmic reticulum (ER) membrane [9]. Released PERK is activated by oligomerization and reverse autophosphorylation, and activated PERK phosphorylates the alpha subunit of translation initiation factor 2 (eIF-2α). In the early stage of the stress response, phosphorylated eIF2α inhibits protein translation and synthesis, reduces the amount of protein folding load in the ER, and thus exerts a protective effect on the cell [10]. Although UPR activation may maintain cell survival, under conditions of severe or persistent ERS, the adaptive response of the UPR fails to eliminate ERS, and the cell is unable to restore ER homeostasis, ultimately leading to the activation of apoptotic signaling pathways [11,12]. With the increase of the time and intensity of the stress response, phosphorylated eIF-2α induces the activation of transcriptional expression of the transcription factor 4 (ATF4), which promotes the apoptosis signaling molecule C/EBP homologous protein (CHOP) expression, which in turn promotes apoptosis, reduces liver function [9,13]. Apoptosis is a physiological and pathological stimulation signal of cells to the environment and a death process that responds to orderly changes caused by changes in environmental conditions or moderate damage [14]. Apoptosis is a strictly controlled process of multiple genes. These genes are very conserved among species, such as the Bcl-2 family and the caspase family [15]. In addition, oxidative stress mediates apoptosis through ERS [16]. Studies have shown that excess APAP causes oxidative stress, ERS, apoptosis; whether FA attenuates these problems is a question to be addressed in this study [17].

*Forsythiae fructus*, a shrub of the genus *Forsythia* in the family *Oleaceae*, is commonly used in the treatment of acute febrile colds and lymph node tuberculosis. The extract of *Forsythia suspensa* has a protective effect against carbon tetrachloride-induced hepatotoxicity. [18,19]. *Forsythia* has a variety of active ingredients, some of which have been proven to have significant pharmacological activity. Forsythiaside A (FA), a phenylethanoid glycoside isolated from *Forsythiae fructus*, exhibits anti-oxidative, anti-inflammatory, and hepatoprotective pharmacological activities [20]. FA has been demonstrated to be involved in regulating the development of a variety of diseases in the body, including inflammation, viral infections, neurodegeneration, oxidative stress, liver injury, and bacterial infections [21,22]. It has been shown that FA plays a protective role in AILI in zebrafish [20]; however, the therapeutic effect of FA in mice with AILI and its mechanism have not been investigated yet.

Understanding the molecular mechanisms of APAP-induced acute liver injury is fundamental to designing new therapeutic strategies for treating this type of liver injury. We presently report that mice given FA exhibit much less liver injury than WT control mice after APAP overdose treatment, suggesting that FA is highly effective for the treatment of APAP-induced acute liver injury. We further demonstrated that FA ameliorates APAP hepatotoxicity metabolism by inhibiting *PXR*-mediated CYP1A2 and CYP3A11. Moreover, *PXR* interference attenuated APAP-induced oxidative stress, ERS, and apoptosis. Ultimately, our study emphasizes that previously undescribed FA alleviates APAP-induced liver dysfunction by inhibiting oxidative stress, ERS, and apoptosis in the liver.

## 2. Results

### 2.1. FA Attenuated APAP-Induced Hepatotoxicity In Vitro, with Optimum Concentration of 20 µM

**Figure 1:**
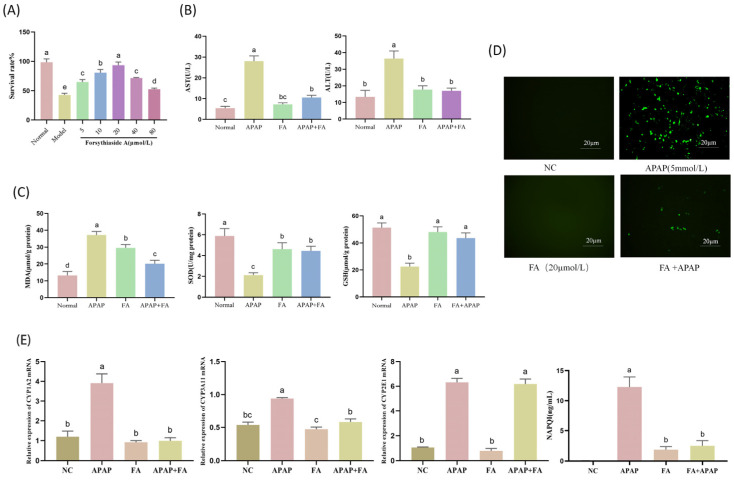
FA therapy for APAP injury in AML12. (**A**) CCK8 detects cellular activities in the APAP model treated with 5, 10, 20, 40, and 80 µmol of FA. Detection of (**B**) ALT, AST, (**C**) MDA, SOD, GSH, and visualization of (**D**) ROS levels after treatment with 20 µmol FA. (**E**) Detection of gene expression levels of APAP metabolism-related enzymes (CYP1A2, CYP3A11, CYP2E1) and APAP metabolite NAPQI content. Different lowercase letters indicate significant differences (*p* < 0.05), while the same lowercase letters indicate no significant differences (*p* > 0.05).

AML12 cells given 5 mM APAP were used as model cells and then given 0, 5, 10, 20, 40, and 80 µM of FA, respectively, to detect the cellular activity. The results, as shown in Figure 1A, show that FA at a concentration of 20 µM had the best effect against APAP liver damage. AST and ALT activities were measured to investigate the effect of FA on AILI. As shown in Figure 1B, AST and ALT activities were significantly elevated in APAP-induced cells compared with the normal group, whereas FA-treated cells had significantly lower AST and ALT activities compared with the model group. This suggests that FA attenuates AILI by inhibiting hepatic transaminase activity. Excess APAP stimulation leads to increased intracellular ROS production in AML12 cells (Figure 1D), resulting in oxidative damage. Depletion of antioxidant indicators such as glutathione and superoxide dismutase led to disruption of intracellular homeostasis and progression of liver injury. To evaluate the antioxidant capacity of FA, the levels of ROS, MDA, SOD, GSH, and NAPQI were determined, and in vitro assays were carried out using doses of 20 µmol/L. As shown in Figure 1C, compared with the normal group, APAP induction significantly increased ROS and MDA levels and reduced GSH and SOD levels. FA treatment significantly upregulated GSH and SOD activity and inhibited excessive ROS and MDA production in AML12 cells stimulated using APAP. These findings showed that FA attenuated APAP-induced oxidative stress in AML12 cells.

To investigate whether the alleviation of APAP hepatotoxicity by FA is related to the inhibition of APAP metabolism level, we examined CYP1A2, CYP3A11, and CYP2E1 mRNA levels in AML12 cells, and the results were as shown in Figure 1E. FA significantly down-regulated the levels of CYP1A2 and CYP3A11, but CYP2E1 seemed to be unaffected. In addition, FA greatly decreased the production of the APAP metabolite NAPQI.

### 2.2. PXR Inhibited by FA In Vitro

**Figure 2:**
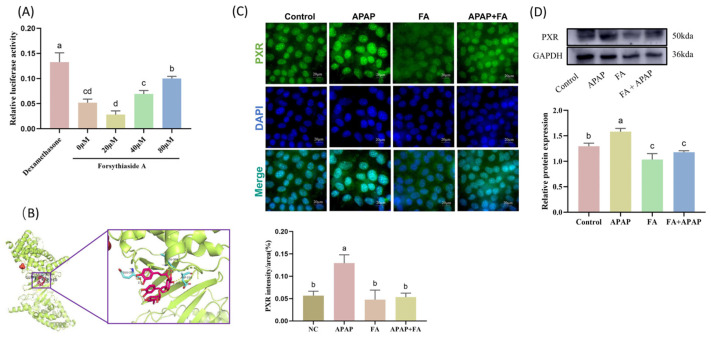
FA inhibits *PXR* expression in AML12 cells. (**A**) 20 µM of FA inhibits activation of the *PXR* promoter. (**B**) Molecular docking of FA and *PXR*. (**C**) Immunofluorescence and (**D**) protein detection of *PXR* expression in AML-12 cells and visualization of results analysis. Different lowercase letters indicate significant differences (*p* < 0.05), while the same lowercase letters indicate no significant differences (*p* > 0.05).

As the important regulator for the treatment of APAP liver injury, we evaluated the possible mechanism of FA in the treatment of AML12 cells under APAP stimulation by detecting the expression of the protein of *PXR*. To determine whether FA affects *PXR*, we constructed a *PXR* promoter reporter gene [23], with *PXR* inducer dexamethasone as a positive control. FA at 0, 20, 40, and 80 μM was given to detect the relative luciferase activity. The results showed that 20 μM FA had the best inhibitory effect on *PXR*, which was consistent with the results of the anti-APAP hepatotoxicity screening (Figure 2A). Meanwhile, using AutoDock 4.2 software, we evaluated the interaction of FA with *PXR* proteins and found a docking energy of 0.97 kcal/mol. Notably, the active ingredient formed hydrogen bonds with the target and had a good binding capacity (Figure 2B). In addition, the expression of *PXR* was detected using WB and immunofluorescence double staining, and compared with the normal group, APAP stimulation increased *PXR* expression, while FA significantly decreased *PXR* expression (*p* < 0.05), and FA was able to reduce cell injury in the APAP-stimulated group (Figure 2C,D).

### 2.3. FA Decreased APAP Metabolism In Vitro by Inhibiting PXR

**Figure 3:**
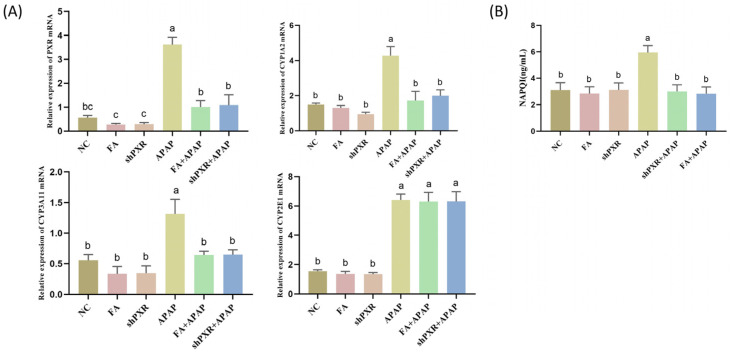
FA reduces APAP metabolism by inhibiting *PXR* in AML12 cells. FA reduced (**A**) CYP1A2 and CYP3A11 mRNA levels and (**B**) APAP metabolite NAPQI content by inhibiting *PXR*. Different lowercase letters indicate significant differences (*p* < 0.05), while the same lowercase letters indicate no significant differences (*p* > 0.05).

To elucidate the mechanism of FA on the protection of APAP against AML12 cell injury, we need to investigate whether FA affects APAP metabolism by inhibiting *PXR*. The mRNA levels of *PXR*-mediated APAP metabolizing enzymes CYP1A2 and CYP3A11 were detected, and the results showed that the APAP group was significantly higher than the normal group. Metabolizing enzyme levels were significantly down-regulated in the FA with the shPXR group compared to the NC. Although CYP2E1 was the main APAP metabolizing enzyme, it was not affected by *PXR* (Figure 3A). In addition, we examined the levels of the APAP metabolite NAPQI, and the results showed that both FA and shPXR significantly reduced its levels, and the effects of FA and shPXR treatment were comparable in the APAP model (Figure 3B).

### 2.4. Inhibition of Oxidative Stress, ERS, and Apoptosis by FA In Vitro

**Figure 4:**
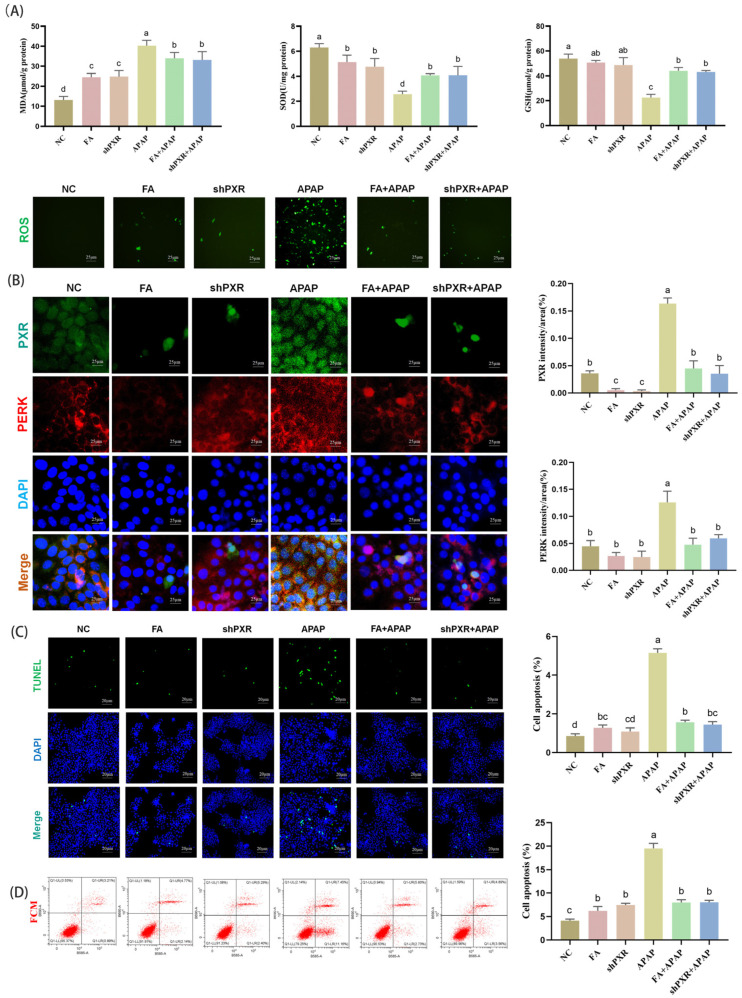
FA alleviates oxidative stress, endoplasmic reticulum stress, and apoptosis in the liver of mice by inhibiting the *PXR*. (**A**) FA down-regulated MDA levels, elevated SOD and GSH levels, and significantly reduced ROS content. (**B**) Inhibition of endoplasmic reticulum stress by FA in mice. Detection of protein expression of *PXR* and PERK by immunofluorescence double staining and its data analysis. Visualization of AML12 cell apoptosis levels using (**C**) TUNEL technology and (**D**) flow cytometry. Different lowercase letters indicate significant differences (*p* < 0.05), while the same lowercase letters indicate no significant differences (*p* > 0.05).

To explore the effects of oxidative stress by FA, we detected the content of ROS in AML12 cells and found that FA could significantly reduce the production of ROS, which was close to the *PXR* interference model. After exposure to APAP, MDA content increased significantly, SOD and GSH decreased significantly, and FA and shPXR groups could restore this abnormal change to normal levels (Figure 4A).

ERS occurs following APAP administration, and several molecules, including PERK, play an essential role in APAP-induced hepatotoxicity. PERK levels were significantly increased in AML12 cells after APAP exposure. However, this increase was completely abolished in cells after *PXR* interference, and the same effect was observed in FA-treated cells (Figure 4B). In addition, combined with the results of immunofluorescence double staining, FA caused a significant decrease in the expression of *PXR*, which was similar to the effect of the shPXR group. Collectively, these data suggest that *PXR* promotes ER stress in response to the presence of hepatotoxic metabolites formed after APAP treatment in AML12 cells.

To investigate the FA effect on apoptosis, we performed TUNEL staining and flow cytometry (FCM). The results showed that the apoptosis rate of cells damaged by APAP was significantly higher than that of the normal group, while the apoptosis rate could be significantly reduced after *PXR* interference treatment or FA treatment (Figure 4C,D).

### 2.5. FA Reduces APAP Metabolism In Vivo by Inhibiting PXR

**Figure 5:**
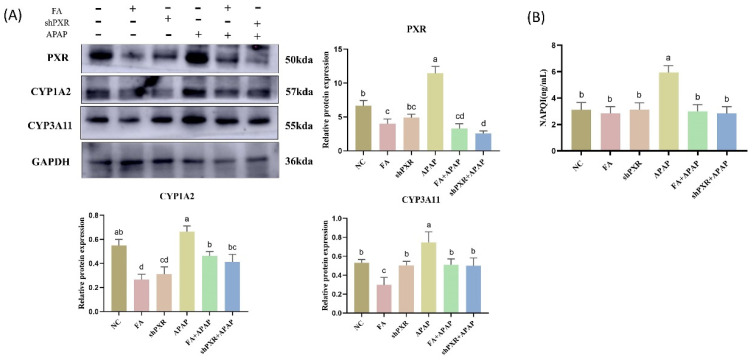
FA treats APAP liver injury in mice by down-regulating *PXR* levels. (**A**) Protein detection of *PXR*, CYP1A2, and CYP3A11 expression in mice and visualization of Western blot results analysis. (**B**) Detection of NAPQI content of APAP metabolite. Different lowercase letters indicate significant differences (*p* < 0.05), while the same lowercase letters indicate no significant differences (*p* > 0.05).

To elucidate the mechanism of FA on the protection of APAP against liver injury, we need to investigate whether FA affects APAP metabolism by inhibiting *PXR* in mice. The protein levels of APAP-metabolizing enzymes mediated by *PXR* were detected. The results showed that the protein levels of CYP1A2 and CYP3A11 in the APAP group were significantly higher than those in the normal group. Metabolizing enzyme levels were significantly down-regulated in the FA with shPXR group compared to the NC (Figure 5A). In addition, we examined the content of the APAP metabolite NAPQI, and the results showed that the NAPQI content was significantly reduced in the APAP model after FA or shPXR treatment (Figure 5B).

### 2.6. FA Plays a Hepatoprotective Role by Inhibiting APAP-Induced Oxidative Stress, ERS, and Apoptosis In Vivo

**Figure 6:**
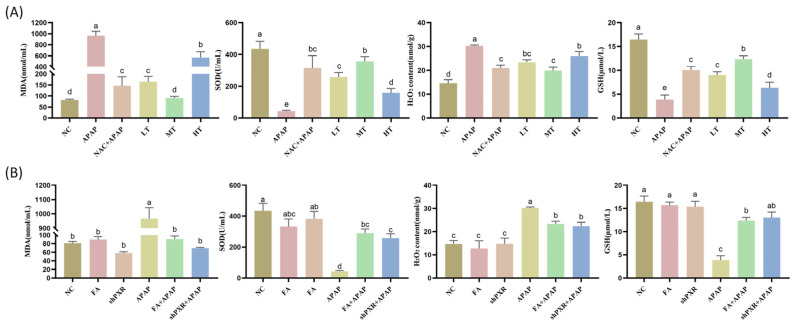
FA reduces oxidative stress levels by inhibiting PXR in mice. (**A**) Detect the effects of FA at different concentrations on the indicators of oxidative stress. (**B**) Detect the effects of the inhibition of PXR on the level of oxidative stress in the APAP injury model. Different lowercase letters indicate significant differences (*p* < 0.05), while the same lowercase letters indicate no significant differences (*p* > 0.05).

In order to investigate the role of *PXR* in the process of FA treating APAP-induced oxidative stress, MDA, SOD, H_2_O_2_, and GSH levels in liver serum were measured. Even though FA at low, medium, and high doses all had a certain alleviating effect on APAP-induced oxidative stress, the medium-dose FA treatment group significantly reduced the contents of MDA and H_2_O_2_, and increased the contents of SOD and GSH, with better effects than the liver-protectant NAC (Figure 6A).

To investigate the effect of FA on oxidative stress in mice, MDA, SOD, H_2_O_2_, and GSH levels in liver serum were measured. As shown in Figure 6B, compared with the normal group, APAP induction significantly increased ROS and MDA levels and reduced GSH and SOD levels. FA treatment significantly upregulated GSH and SOD activity and inhibited excessive ROS and MDA production in APAP model mice. These findings showed that FA attenuated APAP-induced oxidative stress in mice.

**Figure 7:**
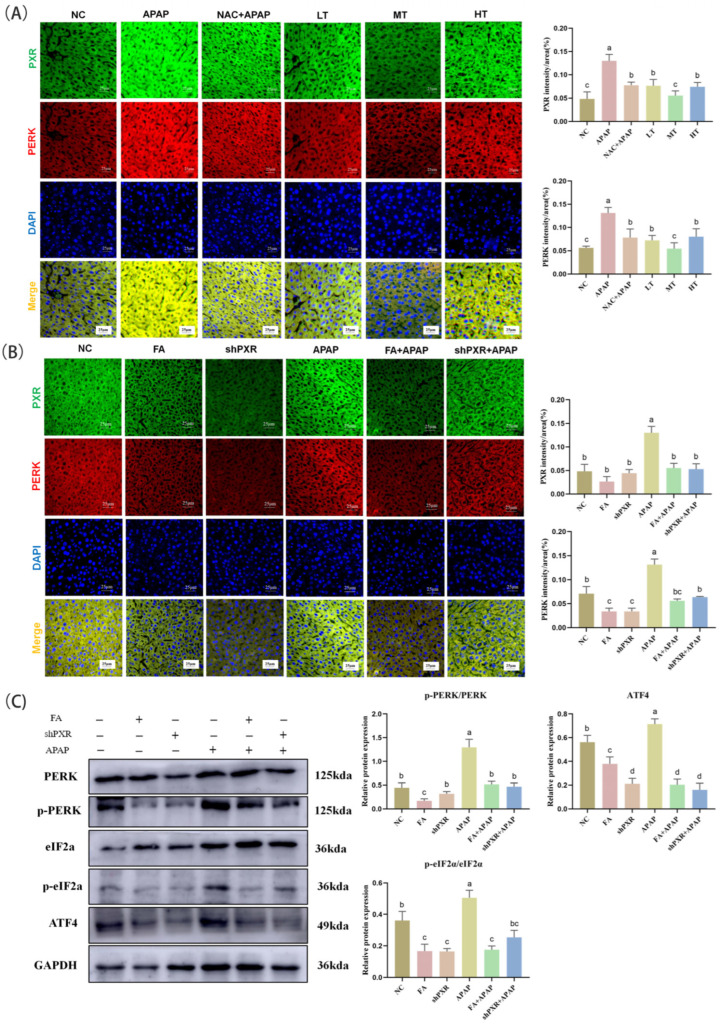
FA reduces endoplasmic reticulum stress levels by inhibiting *PXR* in mice. (**A**,**B**) Immunofluorescence detection of *PXR* and PERK expression in mice and visualization of results analysis. (**C**) Protein detection of PERK, p-PERK, eIF2α, p-eIF2α, and ATF4 expression in mice and visualization of Western blot results analysis. Different lowercase letters indicate significant differences (*p* < 0.05), while the same lowercase letters indicate no significant differences (*p* > 0.05).

To investigate the effect of FA on APAP-induced oxidative stress, the protein expression of the PERK-eIF2α-ATF4 pathway was detected. The results of fluorescence double staining showed that there was a strong correlation between *PXR* and PERK. Although FA at different concentrations could inhibit the expression of PERK, the medium-dose FA had the best effect, which was superior to that of NAC (Figure 7A).

In order to explore the relationship between FA and ERS, we detected PERK-eIF2α-ATF4 pathway factors and their phosphorylated proteins. The results of WB and immunofluorescence double staining were shown in (Figure 7B,C). APAP significantly up-regulated the protein levels of ERS-related factors, while shPXR and FA treatment down-regulated ERS levels.

**Figure 8:**
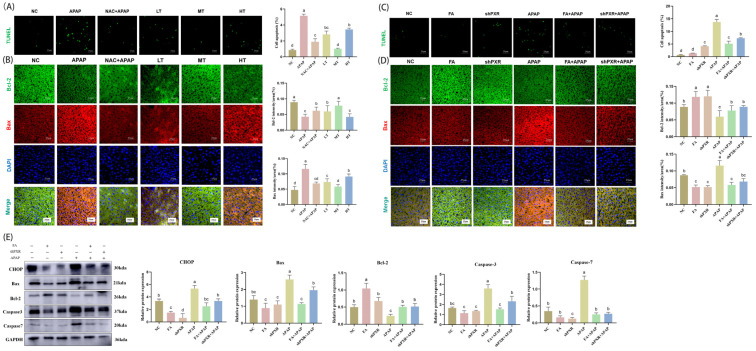
FA inhibits the apoptosis rate by PXR in mice. (**A**,**C**) Visualization of liver cell apoptosis levels using TUNEL technology and calculation of the apoptosis rate. (**B**,**D**) FA reduces Bcl2 and Bax levels by inhibiting PXR in mice. Immunofluorescence detection of Bax and Bcl2 expression, and visualization of results analysis. (**E**) FA reduces CHOP, Bax, Bcl2, Caspase 3, and Caspase 7 levels by inhibiting PXR in mice. The expression of reduced CHOP, Bax, Bcl2, Caspase 3, and Caspase 7 was detected and analyzed by means of Western blot. Different lowercase letters indicate significant differences (*p* < 0.05), while the same lowercase letters indicate no significant differences (*p* > 0.05).

To investigate the effect of FA on APAP-induced apoptosis, TUNEL assay and immunofluorescence were used for detection. The TUNEL results showed that although FA at different doses could reduce the number of apoptotic bodies, the medium-dose FA had the best effect, which was superior to that of NAC (Figure 8A). The immunofluorescence double staining results indicated that although FA at different doses could increase the level of the anti-apoptotic factor Bcl-2 and decrease the level of the pro-apoptotic factor Bax, the medium-dose FA exhibited the optimal effect, outperforming NAC (Figure 8B).

For investigating whether the therapeutic mechanism of FA on AILI was related to apoptosis, we examined the protein expression of Bax, Bcl-2, Caspase 3, and Caspase 7, as shown in Figure 8. The results showed that excess APAP significantly up-regulated the expression of CHOP, Bax, Caspase 3, and Caspase 7, and down-regulated Bcl-2, whereas both shPXR and FA treatments eliminated that increase. Meanwhile, TUNEL staining results showed that the apoptosis rate of liver cells in the APAP group was significantly higher than that in the normal group, while FA and shPXR treatments significantly reduced the high apoptosis rate caused by excess APAP (Figure 8C–E).

**Figure 9:**
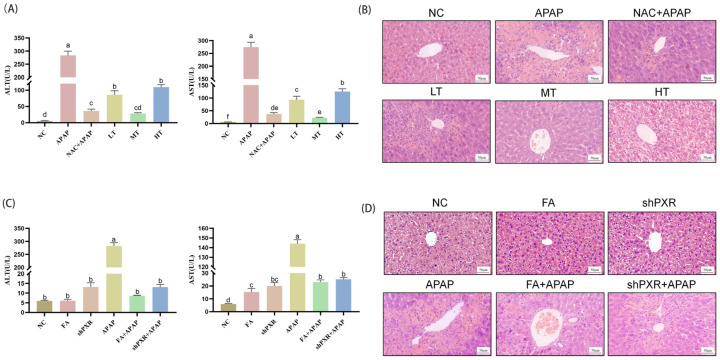
FA attenuates APAP hepatotoxicity in mice. Detection of (**A**,**C**) liver function index ALT and AST, and visualization of (**B**,**D**) liver pathological section from C57 mice. Different lowercase letters indicate significant differences (*p* < 0.05), while the same lowercase letters indicate no significant differences (*p* > 0.05).

To investigate the effects of FA on liver function and liver pathological damage caused by APAP, the levels of ALT and AST were measured, and pathological sections were observed. The results of liver function tests showed that FA at different concentrations could reduce the levels of ALT and AST, but the medium-dose FA had the most significant effect, which was superior to that of NAC (Figure 9A). The results of pathological sections indicated that FA at different concentrations could alleviate liver injury caused by APAP. However, the pathological changes in the medium-dose group were the least, and this group outperformed the NAC treatment group (Figure 9B).

The liver serum biochemical index was measured to investigate the effect of FA on AILI in vivo. Compared with the control group, the levels of ALT and AST in mice increased after exposure to excessive APAP, while FA inhibited the up-regulation of ALT and AST (Figure 9C). In addition, H&E staining showed that the liver tissue morphology of the control group was normal, and the liver cells were intact and arranged neatly. On the contrary, mice exposed to APAP showed uneven nuclear size, nuclear shrinkage, morphological changes, and irregular arrangement (Figure 9D). FA treatment can significantly reduce the changes of liver cells caused by APAP exposure in mice, indicating that FA has a potential hepatoprotective effect.

## 3. Materials and Methods

### 3.1. Cell Experiments

AML12 cells obtained from Novobio Scientific (Shanghai, China) were incubated in DMEM/F-12 medium with 10% FBS at 37 °C and 5% CO_2_ conditions. AML12 cells were inoculated overnight into 6-well plates at a density of 105 cells/well, treated with FA (0–80 μM) for 24 h, and the survival rate of FA was measured using the CCK8 method. To evaluate the therapeutic effects of FA on APAP damage, the cells were pretreated with APAP (5 mM) for 1 h and exposed to FA (20 μΜ) for 24 h. The cell supernatants were collected to detect the levels of oxidative stress and APAP metabolism. And then in order to detect whether FA alleviates APAP damage through PXR, the cells were divided into six groups: normal, APAP, FA, shPXR, FA + APAP, and shPXR + APAP. Short hairpin RNA (shRNA) was purchased from GenePharma (Shanghai, China). Cells were placed in six-well plates and incubated for 24 h before RNA interference. Diluted transfection reagent lip2000 was lightly mixed with shRNA suspension in DMEM (shRNA sequences are detailed in Table 1). Cells were incubated at 37 °C for 6 h; the medium was changed and incubated for 48 h to confirm the infection rate; the medium was changed, and the infection rate was confirmed by incubation for 48 h. The supernatant and cells were collected for subsequent experimental analysis after treatment of the cells.

### 3.2. Animals and Treatment

Male C57BL/6 mice (18–22 g, 6 weeks old) were procured from the Lanzhou veterinary research institute, Chinese academy of agricultural sciences (certificate No. SCXK(J) 2011–0007, Lanzhou, China). The animals were raised in cages and provided free access to food and water under standard specific pathogen-free (SPF) conditions. Animal experiments, including sample collection, were performed in accordance with the guidelines of the Ethics Committee of Gansu Agricultural University. All experiments were approved by the Ethics Committee of Gansu Agricultural University, China (ethics approval file No. GSAU-Eth-VMC-2023-006). All mice were housed on a 12 h light and 12 h dark schedule and fed with deionized water and a standard mouse diet at a suitable temperature (22 ± 0.5 °C). In order to investigate the therapeutic effects of FA at different concentrations on APAP-induced liver injury and to compare the liver-protective effects of FA with those of the liver-protectant NAC (N-acetyl-cysteine), the mice were divided into six groups: the normal group, the APAP group, the NAC treatment group, the low-dose FA treatment group (50 mg/kg, LT), the medium-dose FA treatment group (100 mg/kg, MT), and the high-dose FA treatment group (200 mg/kg, HT). In order to investigate the role of PXR in the process of FA treating APAP-induced liver injury, The mice were divided into six groups: normal group, APAP group, FA group, shPXR group, FA + APAP group, and shPXR + APAP group. Liver and serum were collected for subsequent analysis. APAP solution was made fresh in PBS at 20 mg/mL, and C57BL/6 mice were administered a single dose of 400 mg/kg APAP by intraperitoneal injection [24]. FA solution was prepared in physiological saline, and 100 mg/kg was given by single gavage. Plasmids were injected into mice via the tail vein, and Entranster TM in vivo transfection reagent (Engreen Biosystem Co., Ltd., Beijing, China) was used to deliver plasmids [25].

### 3.3. Drugs and Reagents

Forsythoside A (purity > 99.0%) was purchased from Shanghai yuanye Bio-Technology Co., Ltd. (Shanghai, China). NAC was purchased from Beijing Solarbio Science & Technology Co., Ltd. (Beijing, China). APAP was obtained from Shanghai Aladdin Biochemical Technology Co., Ltd (Shanghai, China). DMEM/F-12 and fetal bovine serum (FBS) were obtained from Gibco Life Technologies (Grand Island, NE, USA), fetal bovine serum (FBS). NAPQI enzyme-linked immunosorbent assay (ELISA) kits were obtained from Shanghai huabang Biochemical Technology Co., Ltd. (Shanghai, China). Terminal deoxynucleotidyl transferase dUTP nick end labeling (TUNEL) apoptosis detection kit and Reactive oxygen species assay kit were obtained from Beyotime Biotechnology (Shanghai, China). The serum enzyme activities of aspartate aminotransferase (AST), alanine aminotransferase (ALT), superoxide dismutase (SOD), malondialdehyde (MDA), hydrogen peroxide (H2O2) and glutathione (GSH) were measured by the commercial quantitative kits (Nanjing Jiancheng Bioengineering Institute, Nanjing, China).

### 3.4. Molecular Docking

Molecular docking technology is a method that predicts the interaction mechanism between small molecule ligands and biological macromolecular receptors (such as proteins and nucleic acids) through computational simulation. It is achieved through the following steps: Conformational Search: Generate possible binding conformations of the ligand and the receptor; Energy Evaluation: Calculate the binding free energy of different conformations; Scoring and Ranking: Rank the candidate conformations according to their affinities. Proteins were semiflexibly docked to the receptor, and the molecular docking was visualized using PyMOL 2.5. The docking was performed using the software AutoDock 4.2. The 2D structure of FA was downloaded from PubChem. The three-dimensional protein data bank (PDB) coordinates for PXR (PDB ID: 6TFI) were retrieved from the RCSB Protein Data Bank.

### 3.5. PXR Promoter Reporter Gene

According to the principles of homologous recombination primer design and the promoter sequence of the mouse *PXR* gene, primers were designed using the homologous recombination method. The purified PCR products and pGL3-basic were digested with two restriction enzymes and then ligated. The ligated *PXR* products were transformed into competent cells, which were then inoculated into LB medium for cultivation. After that, positive clone colonies were picked out, and plasmids were extracted for sequencing. After cell culture and transfection, the dual-luciferase activity assay can be used to detect the activation effect of drugs on *PXR*.ROS detection

The medium was removed, and the cells were washed three times with PBS, followed by incubation with 10 mmol/L DCFH-DA for 20 min at 37 °C. We set the excitation wavelength at 488 nm and the emission wavelength at 525 nm, and directly observed and took photos using a laser confocal microscope.

### 3.6. Histopathological Examination of Liver

Liver tissue samples were fixed in 4% paraformaldehyde for 72 h. For H&E staining, tissue samples were dehydrated, washed, paraffin-embedded, and sectioned at a thickness of 5 μm. H&E staining was completed according to the manufacturer’s instructions.

### 3.7. TUNEL Fluorescence Staining

The TdT-UTP barbed end labeling (TUNEL) kit was purchased from Beyotime Biotechnology (Shanghai) Co., Ltd., (Shanghai, China). The TUNEL assay is performed using a one-step TUNEL kit according to the manufacturer’s instructions. In brief, cells were permeabilized with 0.1% Triton X-100 for 5 min and then incubated with TUNEL assay solution for 1 h at 37 °C, protected from light. FITC-labeled TUNEL-positive cells were imaged under a fluorescence microscope using 488 nm excitation and 530 nm emission. Cells with green fluorescence were defined as apoptotic cells.

### 3.8. Western Blotting (WB)

First, we used RIPA buffer (Solarbio, China) to extract protein from mice liver and cultured cells. Then, protein was separated by SDS-PAGE and transferred onto PVDF membranes (Merck Life Science, Darmstadt, Germany). After that, specific primary antibodies were used to incubate with membranes at 4 °C overnight. The next morning, the membranes were washed for 30 min and incubated with the secondary antibodies at room temperature for 1 h. Protein was visualized by chemiluminescence reagents (YEASRN, Shanghai, China). Band intensities were quantified using Image J 1.54f (National Institutes of Health, Bethesda, MD, USA). In this research, we used GAPDH as the normalization. The primary antibodies and secondary antibodies of Western blot: *PXR* (Proteintech group, Wuhan, China), GAPDH (Affinity Biosciences, Cincinnati, OH, USA), CYP1A2 (Affinity Biosciences), CYP3A11 (Bioss, Beijing, China), CYP2E1 (Bioss), PERK (Affinity Biosciences), p-PERK (Affinity Biosciences), eIF-2α (Bioss), p-eIF-2α (Affinity Biosciences), CHOP (Proteintech group), Bax (Bioss), Bcl-2 (Affinity Biosciences), Caspase 3 (Abmart, Shanghai, China), Caspase 7 (Bioss), Goat Anti-Mouse IgG H&L/HRP antibody (Bioss), Goat Anti-Rabbit IgG H&L/HRP antibody (Bioss).

### 3.9. Quantitative Polymerase Chain Reaction (qPCR)

Cells and mouse livers were collected, and total RNA was extracted with TRIzol reagent and reverse transcribed into cDNA using Hiscript II QRT SuperMix for qPCR (Nanjing Vazyme Biotech Co., Ltd., Nanjing, China). PCR amplification was performed in LightCycler 96 (Roche Diagnostics Products (Shanghai) Co., Ltd., Basel, Switzerland) using SYBR qPCR Master Mix. Primers (Table 2) were synthesized by Sangon Biotech (Shanghai, China) Co., Ltd. and used for PCR. The relative expression levels of *PXR*, CYP1A2, CYP3A11, and CYP2E1 mRNA were calculated by the 2^−ΔΔCt^ method.

### 3.10. Statistical Analysis

The values were expressed as mean ± standard deviation (SD). One-way analysis of variance (ANOVA) was performed using SPSS Statistics 29. *p*-values less than 0.05 were considered statistically significant, and values were expressed as mean ± standard deviation (SD).

## 4. Discussion

*PXR*, a nuclear receptor and major regulator of drug metabolism, is highly expressed in the liver and plays an integral role in the control of exogenous and endogenous metabolism. As a potentially important therapeutic target, it is important to fully understand the biological and physiological properties of *PXR* and its functions. Recently, it has been found that excessive intake of APAP induces aberrant activation of *PXR*, which produces pathogenic effects ultimately resulting in liver injury, whereas this phenomenon is significantly attenuated in *PXR* knockout mice [7,26,27]. These studies demonstrate that *PXR* is an important target for the treatment of APAP liver injury, and *PXR* antagonists may be helpful in reducing APAP-induced hepatotoxicity.

Lately, we have observed that a drug monomer, FA, appears to significantly attenuate overdose AILI; however, it is not clear what mechanistic changes within the liver are occurring that lead to recovery from hepatotoxicity, and the molecular signatures involved remain poorly characterized. FA is the main bioactive index constituent of Forsythia, which has significant hepatoprotective, antioxidant, and other pharmacological effects [28]. In the present research, we confirmed that FA significantly attenuated APAP hepatotoxicity in mice by detecting liver function indexes, oxidative stress indexes, and observing pathological sections. In addition, to explore the optimal therapeutic dose of FA, we conducted investigations using low, medium, and high concentrations of FA, with the NAC treatment group serving as a control. The results demonstrated that 100 mg/kg of FA was the most effective in treating APAP-induced liver injury and outperformed the traditional liver-protectant NAC. This suggests that FA could potentially serve as a new alternative drug for clinically treating liver injury. However, before its clinical application, we need to elucidate the mechanism by which FA treats APAP-induced liver injury.

Since *PXR* is an important target for the treatment of AILI, we considered whether the mechanism of FA treatment of AILI is related to *PXR*. We examined *PXR* levels in APAP liver-injured mice given FA and found that *PXR* expression was significantly correlated with whether FA was given or not. Next, we examined *PXR* expression levels in mice after FA administration alone and showed that FA significantly reduced *PXR* expression. In order to exclude the influence of other factors, we set the shPXR treatment group and FA treatment group as a control, and the results showed that these two groups had the same treatment effect. Combined with the previous studies, we conclude that FA is able to alleviate AILI by inhibiting *PXR*.

In addition, our results demonstrated that FA was able to affect APAP metabolism through inhibition of *PXR*, and after *PXR* was inhibited, CYP1A2 and CYP3A11, two APAP metabolizing enzymes, were inhibited, whereas CYP2E1 was unaltered, which proved that FA did not affect CYP2E1 through *PXR*. Moreover, the metabolite of APAP, NAPQI, was efficiently removed by FA, which explains that FA affects APAP metabolism via *PXR*, thereby ameliorating APAP hepatotoxicity.

Some reports have demonstrated that FA can attenuate ischemic brain injury through the ERS signaling pathway, but the mechanism of treating liver injury is not clear, and the present study fills this gap [29]. After the occurrence of APAP liver injury, the organism will have a series of stress responses, such as ER stress, in order to maintain homeostasis [30]. Preliminary studies in our laboratory have found that drugs for APAP liver injury can significantly reduce the level of ERS, and in conjunction with the study of *PXR* in this experiment, we need to consider whether the alleviation of APAP liver injury caused by FA by inhibiting *PXR* is related to ERS. Our studies at the in vitro and in vivo levels in mice by means of WB and immunofluorescence double staining revealed a positive correlation between *PXR* and PERK expression in all groups and corresponding changes in eIF2α, ATF4, and CHOP. To exclude the influence of other factors, we set up the shPXR + APAP group as a way to demonstrate that the reduction of *PXR* in the APAP model of liver injury can alleviate ER stress, which was confirmed by our data. Previously, we have demonstrated that FA acts as an antagonist of *PXR* in the treatment of APAP hepatotoxicity, and the level of ER stress was significantly decreased in the FA-treated model group, and combined with the above results, we conclude that FA regulates ER stress by affecting the level of *PXR*, which subsequently attenuates liver injury.

Furthermore, ERS can mediate apoptosis, so does it alter apoptosis in a model group treated with FA? We performed the next step of the study. Regarding ERS, we examined the expression of the PERK-eIF2α-ATF4-CHOP pathway and showed that AILI-triggered ERS induced CHOP. The study demonstrated that CHOP is able to cause apoptosis, but it does not mean that all apoptosis that occurs is caused by the CHOP pathway, so we supplementally examined other apoptotic hallmark factors, such as Bax, Bcl-2, Caspase 3, Caspase 7, and flow cytometry with TUNEL staining, which confirmed that excess APAP did trigger apoptosis. The apoptosis rate was significantly decreased after treatment with FA or shPXR, and we finally concluded that FA alleviated ERS-induced apoptosis by inhibiting *PXR*. Previous studies have found that there is a possibility of false positives in gene-level regulation. Therefore, to establish a reliable causal logic, this study designed a rescue experiment. In this experiment, using APAP + *PXR*-overexpressing mice as a control, FA could significantly down-regulate the level of oxidative stress. On this basis, the *PXR* gene was interfered with to investigate whether it could rescue the damage caused by *PXR* overexpression. The results showed that down-regulating *PXR* could rescue the liver injury caused by up-regulating *PXR* (see Figure S1 in the Supplementary Materials), clarifying that the mechanism of FA in treating APAP-induced liver injury is through inhibiting *PXR*-mediated oxidative stress and pathological damage.

However, this study still has certain limitations. In the current paper, the mechanistic depth (*PXR* pathway) has not been fully explored. Although we have preliminarily confirmed that FA can alleviate AILI by inhibiting *PXR*, the more detailed signaling network downstream of *PXR* and its interactions with other intracellular signaling pathways remain unclear. Future research will focus on using cutting-edge technologies such as transcriptomics and proteomics to comprehensively analyze the detailed molecular mechanisms of the *PXR* pathway in AILI. Through transcriptomic analysis, we can gain in-depth insights into the overall changes in gene expression and identify the key genes and signaling pathways regulated by *PXR*; proteomics will help reveal the changes at the protein level and further clarify the interaction relationships between *PXR* and other proteins.

## 5. Conclusions

In conclusion, our study suggests that *PXR* in hepatocytes is a key factor in the initial phase of APAP acute liver failure. We have revealed a key mechanism for the treatment of AILI that is mediated by APAP metabolism by inhibiting *PXR*-mediated CYP1A2 and CYP3A11 via FA. Meanwhile, FA alleviated APAP-induced liver function abnormalities by inhibiting hepatic oxidative stress, ERS, and apoptosis. Our data emphasize FA as a potential therapeutic agent for APAP-induced hepatotoxicity and thus may open new avenues for effective treatment of AILI and its associated complications.

## Figures and Tables

**Table 1:** shRNA sequences.

Carrier Name	pGPU6/GFP/Neo-Nr1i2-Mus-804
Target Sequence	CAAACCTTTGACACAACTTTC
shRNA Template Sequence	S 5′-CACCGCAAACCTTTGACACAACTTTCTTC AAGAGAGAAAGTTGTGTCAAAGGTTTGTTTTTTG-3′
	A 5′-GATCCAAAAAACAAACCTTTGACACAACTT TCTCTCTTGAAGAAAGTTGTGTCAAAGGTTTGC-3′

**Table 2:** Sequences of primers.

Genes	Sequences (5′−3′)
*PXR*	CAACCTACATGTTCAAAGGCATC
	ACACTCCCAGGTTCCAGTCTC
*CYP1A2*	AGTGTTCTGGATGGTCAGAGC
	GCAAGAGGATGCTGACGTCG
*CYP3A11*	AGTGTTCTGGATGGTCAGAGC
	CTCCTTGAGGGAAACTCATGCTCC
*CYP2E1*	CCGCATCCAAAGAGAGGCACAC
	GCACAGCCAATCAGAAAGGTAGGG

## Data Availability

The original contributions presented in this study are included in the article/Supplementary Materials. Further inquiries can be directed to the corresponding author.

## Supplementary Materials

The following supporting information can be downloaded at: https://www.mdpi.com/article/10.3390/molecules30051187/s1, Figure S1: PXR gene rescue experiment in mice.

## Abbreviations

The following abbreviations are used in this manuscript:

APAP	Acetaminophen
AILI	APAP-induced liver injury
FA	Forsythiaside A
ERS	Endoplasmic reticulum stress
PXR	Pregnane X receptor
DILI	Drug-induced liver injury
NAPQI	N-acetyl-p-benzoquinoimide
GSH	glutathione
UPR	unfolded protein response
PERK	ProteinkinaseR-likeERkinase
CHOP	C/EBP homologous protein
TUNEL	TdT-UTP barbed end labeling
