# Supplementary material for: Forsythiaside A Reduces Acetaminophen Hepatotoxic Metabolism by Inhibiting Pregnane X Receptor
Source: Molecules. 2025 Mar 6;30(5):1187. doi: 10.3390/molecules30051187 (PMC11902173; doi:10.3390/molecules30051187)
Supplement: Supplementary file 1 [file molecules-30-01187-s001.zip › Figure S1.pdf]

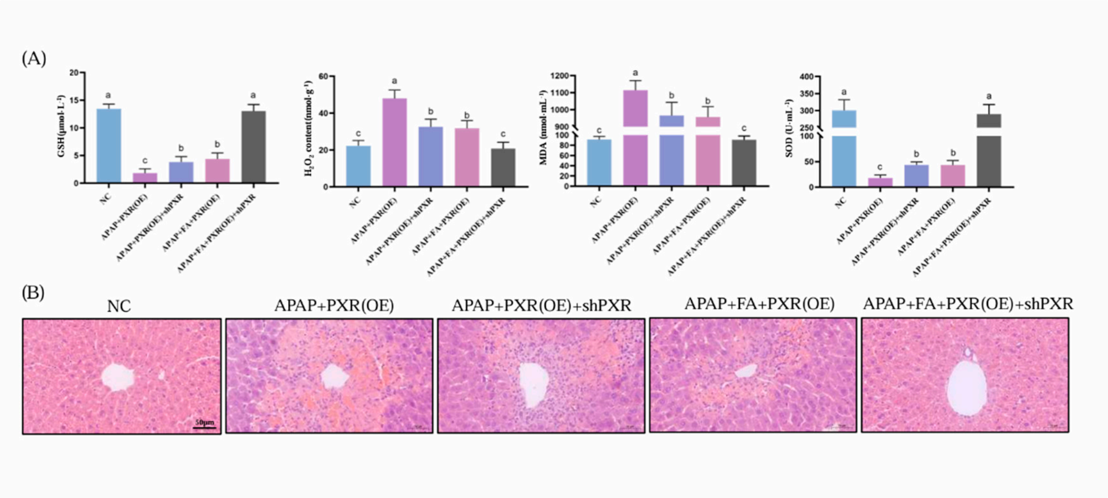

Figure S1. PXR gene rescue experiment in mice. (A) Histopathological changes in the liver tissues of mice with APAP-induced liver injury after PXR interference. (B) Interfering with PXR can reduce the levels of GSH, H_2O_2, MDA, and SOD in the livers of mice. Different shoulder marks indicate significant letter difference ($p < 0.05$), while the same shoulder marks indicate no significant difference ($p > 0.05$).
